# Economic Impact of Food Safety Outbreaks on Food Businesses

**DOI:** 10.3390/foods2040585

**Published:** 2013-12-12

**Authors:** Malik Altaf Hussain, Christopher O. Dawson

**Affiliations:** Department of Wine, Food and Molecular Biosciences, Lincoln University, Lincoln 7647, New Zealand; E-Mail: christopher.dawson@lincoln.ac.nz

**Keywords:** food safety, economic losses, outbreaks

## Abstract

A globalized food trade, extensive production and complex supply chains are contributing toward an increased number of microbiological food safety outbreaks. Moreover, the volume of international food trade has increased to become very large. All of these factors are putting pressure on the food companies to meet global demand in order to be competitive. This scenario could force manufacturers to be lenient toward food safety control intentionally, or unintentionally, and result in a major foodborne outbreak that causes health problems and economic loss. The estimated cost of food safety incidents for the economy of the United States is around $7 billion per year which comes from notifying consumers, removing food from shelves, and paying damages as a result of lawsuits. Most other countries similarly have economic losses. Much of these losses represent lost markets, loss of consumer demand, litigation and company closures. Concrete steps are needed to improve safety of foods produced for local or overseas markets to avoid unexpected food scandals and economic losses.

## 1. Introduction

The potential impact of food safety outbreaks on a food business or a company can be devastating. A single event of a foodborne disease outbreak can bring unimaginable economic losses [1]. An increase in the globalized food trade in recent years, extensive production often involving many sites and a complex supply chain all contribute toward an increased number of microbiological food safety outbreaks. Moreover, the volume of international food trade increases yearly. These are the reasons behind huge pressure on the food companies to be competitive globally and sometimes could result in a relaxed attitude toward food safety control by the producers, eventually causing a food scandal. Economic analysis of food safety related costs showed that it is much cheaper for a producer to invest in preventing events of foodborne outbreaks than the cost after an event [2]. There are many reasons for these companies to improve the safety of their products:

To avoid financial loss due to loss of business.
To avoid unexpected expenses on recalls, disposal, and penalties.
To avoid legal costs due to foodborne outbreaks.
To maintain the reputation of the company.
To maintain consumer’s confidence and loyalty.
To meet government regulations and standards.
To ensure supply of safe food products.
To increase sales and exports.

Food safety is important for the people’s general health and daily life, economic development, and social stability, and the government’s and country’s image [3]. For example recent *Clostridium* contamination of whey protein products put the producer company at the centre of a global food safety scare [4]. The company management and government authorities are trying their best to protect the overall reputation of their global business. This incident highlights a strong need for government regulatory agencies to tackle the challenges in food safety. It is understandable that the private sector’s prime target is to increase profits relative to costs; food safety protection for consumers is dependent on strong government regulations [5]. Therefore, it is reasonable to expect a more reactive than preventative approach from regulators on major food safety scandals rather than addressing the underlying problems. This article will emphasize the economic importance of improved food safety for the companies using some examples of outbreaks.

## 2. Key Food Safety Incidents and Economic Losses

An estimated annual 300,000 hospitalizations and 5000 deaths in the U.S. alone are related to food-borne illnesses; which gives us an idea of the likely economic implications. The cost estimate of food safety incidents to the U.S. economy is around $7 billion which comes from notifying consumers, removing food from shelves, and paying damages as a result of lawsuits. Table 1 lists some of the expensive food outbreaks in the world.

Two recent food-contamination cases which occurred in August 2013 reiterate concerns about the effects of globalization on the food supply [6]. Both outbreaks were linked to international food trade. The first outbreak was the *Cyclospora* parasite in Iowa and Nebraska which originated in a salad mix produced in Mexico by a subsidiary of the Californian based Taylor Farms. The parasite can cause severe stomach illness. This outbreak led to the announcement by the company that it was voluntarily suspending shipments of salad mix and other leafy greens from Mexico until the Food and Drug Administration (FDA, Silver Spring, MD, USA) had resolved the issue.

Secondly an announcement of possible contamination of the *Clostridium botulinum* was linked to a concentrated-whey product distributed to food suppliers in a number of countries by Fonterra (Auckland, New Zealand). This particular bacterium is known to cause botulism, a severe and deadly form of food poisoning. Total financial loss is not yet clear. Initial news reports estimated that the company lost more than $60 million within hours of the announcement of the potential contamination in its products. It has been confirmed that 38 tonnes of whey protein products were contaminated. There was a short-term trade disruption, but it is difficult to predict the longer-term impact. Countries such as China, Russia and Sri Lanka imposed a temporary ban on some dairy products from New Zealand. Government agencies are investigating the reasons for this incident. Fonterra also wisely commissioned an independent inquiry into this incident which is now published and available on the Fonterra website [4]. In the authors’ view this is an impressive report that is likely to become an essential reference on handling such possible outbreaks [4]. More recently Danone (Paris, France), one of the customers who received food safety alert, is seeking around €200 million ($270 million) in compensation from Fonterra to cover the costs associated with the infant formula product recalls as a result of the botulism scare [7].

The impact of this incident on small food companies based in New Zealand is much more severe; either their business suffered badly or is likely to be forced to close down due to lack of sales [8]. Apart from this recent food scare in New Zealand, the overall economic cost of foodborne disease in New Zealand was reviewed by Gadiel [9]. It is important that small producers understand the need to have a risk assessment and a plan of how they will handle such an incident. In the authors’ view this would most likely include independent experts.

**Table 1:** World examples of some expensive food outbreaks/recalls [4,10].

Year	Contamination/Food Product	Estimated Economic Loss	Region/Country
2013	*Clostridium botulinum*/Whey concentrate	Unknown	New Zealand
2009	*Salmonella*/Peanut products	$70 million	USA
2008	*Salmonella*/Tomatoes	$250 million	USA
2008	Mad cow disease/Meat	$117 million	USA
2007	*Salmonella*/Peanut butter	$133 million	USA
2006	*E. coli*/Spinach	$350 million	USA
1992	*E. coli*/Hamburgers	$160 million	USA

Two other examples of expensive and devastating food safety incidents were noted in 2008 and 2009 [10]. A deadly outbreak of *Salmonella* food contamination was recorded in 2009 which originated from a plant belonging to the Peanut Corporation of America (PCA, Lynchburg, VA, USA). The outbreak resulted in over 700 cases of serious illness and at least nine deaths. Within days, a second plant was also implicated in contamination, resulting in PCA permanently discontinuing operations. In addition to the economic cost to PCA other food companies were affected. The food giant Kellogg (Battle Creek, MI, USA), announced losses of $70 million related to recall-related losses and “Forward Foods of Minden, NV who make the Detour brand energy bars, was forced into bankruptcy”.

Westland/Hallmark Meat Packing Company (Chino, CA, USA) issued the largest meat recall in the U.S. history in 2008. At the request of the FDA, Westland/Hallmark recalled more than 143 million pounds of beef. The cost of the recall to the company was over $117 million. In November 2012, Westland/Hallmark agreed to settle $500 million on numerous affected customers and the federal government. Before paying, the company was declared bankrupt. Recalls of this scale are rare but will be increasingly frequent as global distribution supply chains improve, detection/sampling techniques improve and regulated allowable limits for contamination fall.

Of late, food recalls have become very common. Using information from the U.S. and Canada, there were more than 600 recalls during the past 12 months [11]. This massive number of recalls indicates how often foods leave the manufacturer’s premises with a potential to cause serious health risks to the consumer. Therefore food recalls are the method by which companies and government regulators try to improve food safety by removing products from distributor inventories, store shelves and consumers’ kitchens. The major reasons behind food products recall are recorded as contamination, adulteration, misbranding and a compromised product. Contamination with a pathogen such as *Listeria monocytogenes*, or presence of undeclared allergens such as peanut, can be quoted as examples. Most of these recalls are precautionary and incur huge costs to companies’ operating expenses. However, recall events are important for the manufacturer to maintain and regain consumer confidence on suspicion of contamination in its products. Moreover, these are essential tools to allow government agencies to remove potentially harmful products from the market rapidly and efficiently in order to protect consumers and general public health.

## 3. Conclusions

The current situation highlights the need for food companies to increase the microbiological safety of the foods that are produced for local or overseas markets to avoid unexpected food safety scares. The economic implications of an outbreak are very serious and may cause the closure of an individual company and long lasting damage to a particular sector of the food industry. Therefore it is important for food companies to:

Understand global food safety issues.
Monitor changing conditions in business.
Consider the effect of transporting on foods.
Promote food safety by working with governmental agencies and professional associations.
Develop food safety awareness.
